# Occupational factors and subsequent major depressive and generalized anxiety disorders in the prospective French national SIP study

**DOI:** 10.1186/s12889-015-1559-y

**Published:** 2015-02-28

**Authors:** Isabelle Niedhammer, Lucile Malard, Jean-François Chastang

**Affiliations:** INSERM UMRS 1136 - IPLESP, Team 7 (ERES), Faculté de Médecine Pierre et Marie Curie - pôle Saint-Antoine, 27 rue de Chaligny, F-75012 Paris, France; Sorbonne Universités, UPMC Univ Paris 06, UMR_S 1136, Pierre Louis Institute of Epidemiology and Public Health, Department of social epidemiology, Paris, F-75013 France; Université de Versailles St-Quentin, Paris, France

**Keywords:** Psychosocial work factors, Occupational factors, Frequency of exposure, Repeated exposure, Depression, Anxiety, Diagnostic interview, Prospective data

## Abstract

**Background:**

The literature has been extensive on the associations between psychosocial work factors and mental health. Nevertheless, the studies using prospective design, various concepts and more than one measurement point in time for these factors and diagnostic interview to assess mental disorders remain seldom in the literature. This study is an attempt to fill the gap in this topic.

**Methods:**

The study was based on a national representative sample of 4717 workers of the French working population (SIP survey), interviewed in 2006 and reinterviewed again in 2010 and free of mental disorders at baseline. Psychosocial work factors, measured in both 2006 and 2010, included: psychological demands, decision latitude, social support, reward, emotional demands, role conflict, ethical conflict, tensions with the public, job insecurity and work-life imbalance. Other occupational factors related to working time/hours and physical work environment were also studied. Major depressive (MDD) and generalized anxiety disorders (GAD) were measured using a standardised diagnostic interview (MINI). Covariates were age, occupation, marital status, having a child under 3 y, social support outside work and stressful life events. Multivariate analyses were performed using weighted logistic regression models.

**Results:**

Using models taking all occupational factors into account simultaneously, low reward and job insecurity predicted MDD. Psychological demands, low reward, emotional demands and job insecurity were predictive of GAD. The more frequent the exposure to job insecurity, the higher the risk of MDD and GAD, and the more frequent the exposure to psychological demands and low reward, the higher the risk of GAD. No effect was observed for repeated exposure to occupational factors.

**Conclusions:**

Classical and emergent psychosocial work factors were predictive factors of depression and anxiety with dose–response associations in terms of frequency of exposure. More attention may be needed on emergent psychosocial work factors and frequent exposure to these factors.

## Background

Mental disorders, and among them the two most common disorders of depression and anxiety, are an important issue in occupational health because of the high costs and heavy impact on absenteeism, presenteeism, and other work-related outcomes such as reduced work performance and turnover [1,2]. Improving the knowledge on occupational risk factors for mental disorders is therefore crucial. Some psychosocial work factors have been identified as risk factors for common mental disorders or mental health outcomes in reviews or meta-analysis of prospective studies [3-7], these studies being often limited to well-known factors or classical factors such as those related to the job strain and effort-reward imbalance models [8,9]. Thus, these reviews and meta-analysis demonstrated that the risk of mental health outcomes, especially depression or depressive symptoms, may increase with high psychological demands, low decision latitude (comprising low skill discretion and low decision authority), the combination of high demands and low latitude, and low social support (job strain model), and with the combination of high effort and low reward (effort-reward imbalance model). The literature appears more seldom for other factors not covered by these two models that we may call emergent factors, and there is a need to explore the psychosocial work environment more widely [4].

Although the reviews and meta-analysis quoted above [3-7] were based on prospective studies, they included all studies whatever the method used to measure mental health outcomes. Consequently, their results may be dependent of the type of outcome studied and the method used to measure it, and the consistency in the results may be altered by differences between studies. Bonde [3] reported that major depression was defined by clinical criteria in less than half of the prospective studies included in his review and concluded that this limitation with other methodological limitations may preclude causal inference. Furthermore, self-reporting of both exposures and mental health outcome (through mental health symptom scales) may lead to exposure misclassification and reporting bias. The use of diagnostic interview to measure mental disorders and the collection of several measures of exposures at different points in time may contribute to overcome these difficulties. In addition, anxiety remains understudied in comparison to depression, and more information may be needed for this disorder. Finally, there is a major need for information regarding dose–response associations between psychosocial work factors and mental disorders, in other words, studies exploring the frequency, intensity or duration of exposure to psychosocial work factors in association with mental disorders remain seldom in the literature [4]. To conclude, studies combining these different strengths, prospective design, exploration of various psychosocial work factors, study of both depression and anxiety, use of diagnostic interview, several measurement points of exposure and study of dose–response associations are still missing.

The objectives of this study were to explore the prospective associations between well-know and understudied psychosocial work factors, and other occupational factors, and the incidence of two mental disorders, depression and anxiety, measured using a diagnostic interview. The study also aimed at examining the associations between the frequency and duration of exposure to these factors and the two outcomes in a national representative sample of French workers.

## Methods

### Sample

The study was based on the data from the prospective national representative SIP (Santé et Itinéraire Professionnel) survey, performed by the French Ministries of Labour and Health (DARES and DREES), the French Centre for Employment Studies (CEE) and the French National Institute for Statistics and Economic Studies (INSEE). This survey was designed to explore the complex associations between work and health [10]. In 2006, households were randomly selected from the 1999 census, that was updated for new housings, and one individual aged between 20 to 74 years was randomly selected to be interviewed in each household. Finally, 13648 men and women from the general French population were interviewed by a trained interviewer at respondent’s home. The participation rate was 76%. Four years later, they were contacted again for the second wave of the survey, and 11016 individuals participated (i.e. a follow-up rate of 81%). Among them, 5116 were working in both 2006 and 2010. Workers having a diagnostic of major depressive disorder (MDD) and/or generalized anxiety disorder (GAD) in 2006 were excluded from the study, i.e. 399 workers. Thus, the study sample included 4717 workers, 2389 men and 2328 women, free of both disorders in 2006 and who were working and followed up from 2006 to 2010. Four studies by our team have already been published using these data [11-14]. One of these studies explored the associations between psychosocial work factors and depression and anxiety using the 2006 cross-sectional data of the SIP survey. The present study is thus an attempt to improve our knowledge of these associations using the prospective data of the SIP survey. The SIP survey was approved by the French Ethics Committees (CNIL and CNIS).

### Mental disorders

The Mini International Neuropsychiatric Interview (MINI) is a structured diagnostic interview for 17 major psychiatric disorders based on the criteria of the Diagnosis and Statistical Manual of Mental Disorders, 4th edition (DSM-IV) [15]. The interviews for the SIP survey in both 2006 and 2010 included the MINI for the diagnosis of MDD and GAD. The time frame for current MDD was the last 2 weeks and for current GAD it was the last 6 months. The validity of the MINI was demonstrated for the French and English versions according to the CIDI and SCID-P as gold standards [16,17]. Consequently, in this study, the two binary outcomes were MDD and GAD.

### Occupational factors

The occupational factors studied were 10 psychosocial work factors, 3 factors related to working time/hours and 3 physical work factors. They were all measured in 2006 and 2010.

Psychosocial work factors were measured following the classical job strain [8] and effort-reward imbalance models [9], and emergent concepts. Three factors or proxies for the dimensions of the job strain model were constructed: psychological demands (3 items: working under pressure, too many things to do, excessive amount of work), decision latitude (2 items: freedom to decide how to do the work, use of skills) and social support (1 item: good relationships with colleagues). One proxy for the reward dimension of effort-reward imbalance model was constructed: reward (1 item: fair feedback on the work done). Emergent psychosocial work factors included: emotional demands (1 item: hiding feelings at work), role conflict (1 item: not being able to work following best practices), ethical conflict (1 item: exposure to unethical situations), tensions with the public (1 item: tensions with the public, users, students, patients, customers), job insecurity (1 item: fear of job loss), and work-life imbalance (1 item: work in line with family life).

Four factors related to working time/hours were studied: long working hours (1 item: working more than 48 hours a week, following the European Directive on working time), night work (1 item: working between midnight and 5 am), shift work (1 item: working on alternating shift), and low predictability (1 item: irregular hours difficult to predict).

Physical work factors included biomechanical exposure (3 items: manual materials handling, other biomechanical constraints -long standing, crouching, bending, arms above the shoulders, or force position-, and vibrations), physical exposure (2 items: loud noise -unable to hear someone who is 2 or 3 m away, even if the person shouts-, and extreme temperatures -exposure to heat, cold, humidity or dirtiness-), and chemical exposure (1 item: exposure to dust, fume, chemical products or germs).

For all items, the response categories were always/often/sometimes/never. Items were summed when the factors included more than one item. Three measures of exposure were used:

- binary variables in 2006: exposed versus non-exposed using the median cut-offs,
- frequency variables in 2006: using the initial response categories, always/often/sometimes/never, for the factors based on one item or quartiles for those with more than one item, and
- repeated exposure evaluated using both the binary variables in 2006 and 2010 for each occupational factor.

### Covariates

The covariates were age, occupation (managers/professionals, associate professionals/technicians, clerks/service workers and manual workers), marital status (living with or without a partner), having a child under 3 years old, social support outside work (4 items: having someone to rely on to discuss personal issues or take a difficult decision -besides partner-, having someone to help on daily tasks, like do-it-yourself or child care, or to borrow some objects, and for each of these two items, need more help than help received), life events during childhood (12 items: disability, long illness, serious health problems or death of close family member, etc.), and life events between 2002 and 2006 (4 items: separation, care or death of close family member, strong deterioration in living conditions). Occupation was coded using the French national classification of occupations that is close of the international classification (ISCO). Covariates were measured in 2006.

### Statistical analyses

To be representative of the French working population of 2006, weights were calculated using marginal calibration and inverse probability weighting to control for the biases due to non-response in 2006 and attrition in 2010 [18]. A marginal calibration on age, work status (working/unemployed/non-working) × age, urban area, size of household, occupation and economic activity was performed on the sample in 2006. Homogeneous response groups were formed based on characteristics in 2006 (work status, urban area, age, level of education, gender and self-reported health), and the probability of being interviewed in 2010 was calculated for each group. Weights calculated by marginal calibration (for non-response in 2006) were multiplied by the inverse probability of being interviewed in 2010. Finally, a second marginal calibration on territorial unit, urban area, age × gender, education, nationality, and size of household was performed on the sample of individuals interviewed in 2006 and 2010 to be representative of the population of 2006. Weights were included in all statistical analyses.

A description of the study sample was done for all studied variables and comparisons between genders were performed using Rao-Scott Chi-Square test.

The prospective associations between occupational factors and mental disorders were studied using the data of 2006 (occupational factors and covariates) and 2010 (outcomes) among the sub-sample of those who were working in both 2006 and 2010 and were free of both mental disorders in 2006. Multivariate weighted logistic regression models were performed to adjust for covariates, MDD/GAD being the dependent variable, among the whole sample of men and women. Two types of models were performed: (i) each occupational factor was introduced separately in the models with adjustment for covariates (models 1), and (ii) all occupational factors were introduced simultaneously with adjustment for covariates (model 2). Although occupational factors displayed interrelations, no collinearity was detected in model 2. In model 2 using binary occupational variables, an interaction term between psychological demands and decision latitude was introduced to test Karasek’s hypothesis of job strain, but was found to be non-significant. Interaction terms were also tested one by one in model 2 between gender and each occupational factor using binary variables to explore potential differences in the associations between genders.

To explore dose–response associations, prospective analyses were performed using the frequency of exposure and repeated exposure to occupational factors. The analysis of the frequency of exposure was done using the frequency variables as continuous variables and trend tests were performed to explore potential linear associations between frequency of exposure and outcome. The analysis of repeated exposure to occupational factors was performed using the exposures of 2006 and 2010 (binary variables), and an interaction term between these two exposures was added to test whether the effect of exposure in 2010 was the same or different according to the values of the exposure in 2006.

Sensitivity analyses were performed including additional covariates that were: employment variables (economic activity of the company, public/private sector, employee/self-employed worker status), overcommitment at work as a personality factor, job change and unemployment/inactivity periods between the two data collections in 2006 and 2010, health status variables (long-standing illness, chronic diseases, work injury, disability, body mass index) and life events between 2006 and 2010. Finally, a sensitivity analysis including all people at follow-up whatever their working status in 2010 (and not only those who were working) was also performed.

All statistical analyses were carried out using SAS 9.3 software (SAS Institute Inc, Cary, NC).

## Results

The description of the study sample is presented in Table 1. Differences in covariates were observed between genders. Men were more likely to be professionals/managers and manual workers, and to have children of 3 y or less. Women were more likely to be clerks/service workers, to live alone, to have low social support outside work and recent stressful life events. Differences in psychosocial work factors were found according to gender. Men were more likely to be exposed to high psychological demands and ethical conflict, and women were more likely to be exposed to emotional demands and tensions with the public. Men were more likely to be exposed to all other factors related to working time/hours and physical work environment. Among the study sample of workers who were free of MDD and GAD in 2006, the incidence of new cases of MDD and GAD in 2010 was higher for women than for men.

**Table 1:** **Description of the study population according to covariates and occupational factors in 2006 and incidence of mental disorders in 2010**

	**Men (N = 2389)**			**Women (N = 2328)**			
	**n**	**%**	**%w**	**n**	**%**	**%w**	**p**
**Covariates in 2006**							
Age (years)							ns
<30	286	11.97	17.94	257	11.04	17.64	
30-39	701	29.34	31.71	647	27.79	28.57	
40-49	832	34.83	32.79	866	37.20	33.26	
≥50	570	23.86	17.56	558	23.97	20.53	
Occupation							***
Managers/professionals	401	16.80	16.66	318	13.67	11.86	
Associate professionals/technicians	602	25.22	25.71	648	27.85	26.38	
Clerks/service workers	327	13.70	17.30	1121	48.17	50.24	
Manual workers	1057	44.28	40.13	240	10.31	11.53	
Living alone	528	22.10	20.30	678	29.12	23.70	*
Presence of child(ren) under 3 y	289	12.10	14.88	200	8.59	10.67	***
Low social support outside work	626	26.20	26.43	739	31.74	29.81	*
Life events during childhood (one or more)	1077	45.08	45.80	1122	48.20	48.73	ns
Life events 2002–2006 (one or more)	207	8.67	8.49	252	10.83	10.76	*
**Occupational factors in 2006**							
**Psychosocial work factors**							
High psychological demands	998	41.77	41.80	840	36.08	35.66	***
Low decision latitude	780	32.65	35.15	728	31.27	32.49	ns
Low social support	565	23.65	23.95	506	21.74	21.93	ns
Low reward	716	29.97	31.12	732	31.44	31.64	ns
Emotional demands	740	30.98	33.43	1084	46.56	46.45	***
Role conflict	1084	45.37	46.86	1066	45.79	45.43	ns
Ethical conflict	857	35.87	35.92	654	28.09	27.86	***
Tensions with the public	959	40.14	41.83	1080	46.39	46.75	**
Job insecurity	520	21.77	22.22	468	20.10	21.10	ns
Work-life imbalance	750	31.39	32.64	693	29.77	29.83	ns
**Working time/hours**							
Long working hours	1008	42.19	42.41	523	22.47	21.94	***
Night work	595	24.91	25.77	230	9.88	10.44	***
Shift work	414	17.33	17.96	322	14.26	15.35	*
Low predictability	794	33.24	34.19	526	22.59	23.66	***
**Physical work factors**							
Biomechanical exposure	1040	45.53	42.89	718	30.84	32.38	***
Physical exposure	1242	51.99	49.63	597	25.64	26.33	***
Chemical exposure	1104	46.21	44.07	579	24.87	26.56	***
**Mental disorders in 2010**							
MDD	61	2.55	2.53	114	4.90	4.75	***
GAD	66	2.76	2.81	120	5.15	4.58	**

%: raw frequency.

%w: weighted frequency.

p: Rao-Scott χ^2^ test p-value for the comparison between genders. ***p < 0.001, **p < 0.01, *p < 0.05.

Table 2 presents the results of multivariate analyses using binary occupational variables. For both models 1 (each occupational factor studied separately) and model 2 (all factors studied simultaneously), low reward and job insecurity were found to be predictive factors of MDD. No interaction was observed with gender. For models 1, all psychosocial work factors, except decision latitude and role conflict, were predictive factors of GAD. Using model 2, psychological demands, low reward, emotional demands and job insecurity predicted GAD. An interaction was found between gender and job insecurity, showing that job insecurity predicted GAD for men (OR = 2.49, 95% CI:1.37-4.55), but not for women.

**Table 2:** **Prospective associations between occupational factors (binary variables) in 2006 and MDD/GAD in 2010 adjusted for covariates**

**Men and women (N = 4711)**	**MDD**				**GAD**			
	**Models 1**		**Model 2**		**Models 1**		**Model 2**	
	**OR** ^**†**^	**95% CI**	**OR** ^**††**^	**95% CI**	**OR** ^**†**^	**95% CI**	**OR** ^**††**^	**95% CI**
**Psychosocial work factors**								
High psychological demands	1.27	[0.85-1.91]	1.13	[0.68-1.86]	2.34***	**[1.64 -3.36]**	1.78**	**[1.20-2.64]**
Low decision latitude	1.38	[0.95-2.00]	1.24	[0.86-1.81]	1.29	[0.89 -1.86]	1.07	[0.73-1.56]
Low social support	1.04	[0.68-1.59]	0.94	[0.58-1.55]	1.48*	**[1.01 -2.18]**	1.08	[0.74-1.59]
Low reward	1.67**	**[1.16-2.40]**	1.60*	**[1.08-2.39]**	1.85***	**[1.32 -2.60]**	1.53*	**[1.06-2.20]**
Emotional demands	1.16	[0.79-1.71]	1.10	[0.73-1.67]	2.14***	**[1.51 -3.04]**	1.66**	**[1.14-2.40]**
Role conflict	0.98	[0.67-1.44]	0.81	[0.53-1.24]	1.29	[0.91 -1.84]	0.88	[0.60-1.29]
Ethical conflict	1.01	[0.68-1.50]	0.91	[0.59-1.40]	1.42*	**[1.00 -2.02]**	0.98	[0.67-1.44]
Tensions with the public	0.77	[0.53-1.12]	0.70	[0.47-1.05]	1.58**	**[1.12 -2.24]**	1.23	[0.85-1.77]
Job insecurity	1.76**	**[1.17-2.64]**	1.63*	**[1.08-2.48]**	2.16***	**[1.47 -3.18]**	1.74**	**[1.18-2.57]**
Work life imbalance	1.09	[0.72-1.63]	1.01	[0.63-1.60]	1.80**	**[1.26 -2.59]**	1.33	[0.90-1.96]
**Working time/hours**								
Long working hours	1.19	[0.80-1.78]	1.22	[0.78-1.91]	1.33	[0.91 -1.94]	1.05	[0.69-1.60]
Night work	1.04	[0.62-1.74]	0.90	[0.52-1.57]	0.89	[0.52 -1.52]	0.85	[0.50-1.45]
Shift work	1.24	[0.77-1.99]	1.25	[0.75-2.07]	0.95	[0.58 -1.56]	0.92	[0.57-1.48]
Low predictability	1.11	[0.75-1.66]	1.06	[0.71-1.59]	1.10	[0.75 -1.62]	0.85	[0.56-1.27]
**Physical work factors**								
Biomechanical exposure	1.17	[0.80-1.70]	1.11	[0.72-1.71]	1.06	[0.73 -1.55]	0.87	[0.59-1.28]
Physical exposure	1.17	[0.79-1.72]	1.07	[0.70-1.65]	1.15	[0.79 -1.66]	0.92	[0.62-1.35]
Chemical exposure	0.92	[0.63-1.33]	0.85	[0.55-1.31]	1.14	[0.79 -1.64]	1.11	[0.78-1.58]

The binary variables for occupational factors were constructed using the median of the distribution among the whole sample.

Weighted logistic regression models.

Covariates: gender, age, occupation, marital status, having a child under 3 y, social support outside work and stressful life events.

^†^Each occupational factor studied separately ^††^All occupational factors studied simultaneously.

Bold OR: significant at 5%.

Wald test: ***p < 0.001, **p < 0.01, *p < 0.05.

Table 3 presents the results using frequency variables for occupational factors. The risk of MDD increased with the frequency of exposure to psychological demands, low reward and job insecurity in models 1, and the linear association between the frequency of exposure to job insecurity and MDD was also observed in model 2. In models 1, all psychosocial work factors, except decision latitude and role conflict, displayed linear associations between frequency of exposure and GAD. The frequency of exposure to psychological demands, low reward and job insecurity increased the risk of GAD in model 2.

**Table 3:** **Prospective associations between occupational factors (frequency variables as continuous variables) in 2006 and MDD/GAD in 2010 adjusted for covariates**

**Men and women (N = 4711)**	**MDD**				**GAD**			
	**Models 1**		**Model 2**		**Models 1**		**Model 2**	
	**OR** ^**†**^	**95% CI**	**OR** ^**‡**^	**95% CI**	**OR** ^**†**^	**95% CI**	**OR** ^**‡**^	**95% CI**
**Psychosocial work factors**								
High psychological demands	1.22*	**[1.01-1.46]**	1.15	[0.92-1.44]	1.69***	**[1.43-1.99]**	1.48***	**[1.22-1.80]**
Low decision latitude	1.14	[0.99-1.32]	1.09	[0.93-1.27]	1.08	[0.94-1.24]	0.98	[0.84-1.14]
Low social support	1.16	[0.88-1.53]	1.05	[0.77-1.44]	1.48**	**[1.16-1.89]**	1.28	[0.99-1.66]
Low reward	1.20*	**[1.01-1.42]**	1.14	[0.93-1.39]	1.33***	**[1.14-1.56]**	1.22*	**[1.01-1.46]**
Emotional demands	1.10	[0.94-1.27]	1.05	[0.89-1.24]	1.32***	**[1.13-1.53]**	1.15	[0.97-1.38]
Role conflict	1.07	[0.84-1.37]	0.92	[0.68-1.24]	1.15	[0.94-1.41]	0.87	[0.70-1.10]
Ethical conflict	1.10	[0.83-1.47]	0.96	[0.69-1.32]	1.39*	**[1.08-1.79]**	1.03	[0.77-1.37]
Tensions with the public	0.90	[0.69-1.18]	0.80	[0.61-1.05]	1.26*	**[1.05-1.52]**	1.06	[0.86-1.30]
Job insecurity	1.45***	**[1.18-1.80]**	1.37**	**[1.10-1.72]**	1.63***	**[1.34-1.98]**	1.46***	**[1.18-1.80]**
Work life imbalance	1.06	[0.84-1.33]	0.94	[0.72-1.23]	1.32**	**[1.10-1.59]**	1.11	[0.90-1.35]
**Working time/hours**								
Long working hours	1.09	[0.91-1.30]	1.09	[0.90-1.33]	1.14	[0.97-1.34]	1.02	[0.84-1.23]
Night work	1.09	[0.84-1.41]	1.02	[0.79-1.32]	0.86	[0.66-1.12]	0.84	[0.64-1.10]
Shift work	1.10	[0.92-1.30]	1.09	[0.91-1.29]	0.97	[0.81-1.17]	0.98	[0.81-1.18]
Low predictability	1.07	[0.88-1.30]	1.02	[0.84-1.25]	1.09	[0.90-1.33]	0.94	[0.76-1.16]
**Physical work factors**								
Biomechanical exposure	1.10	[0.94-1.29]	1.07	[0.89-1.30]	1.04	[0.89-1.20]	0.95	[0.80-1.14]
Physical exposure	1.08	[0.93-1.26]	1.04	[0.87-1.23]	1.04	[0.90-1.20]	0.94	[0.79-1.11]
Chemical exposure	0.97	[0.81-1.17]	0.90	[0.73-1.11]	1.09	[0.93-1.29]	1.08	[0.91-1.28]

The frequency variables for occupational factors were based on the initial coding for the factors with one item or quartiles for the factors with more than one item.

Weighted logistic regression models.

Covariates: gender, age, occupation, marital status, having a child under 3 y, social support outside work and stressful life events.

^†^Each occupational factor studied separately. ^‡^All occupational factors studied simultaneously.

OR associated with an increase of 1 unit of frequency variable.

Bold OR: significant at 5%.

Trend test: ***p < 0.001, **p < 0.01, *p < 0.05.

Table 4 shows the results for the analysis of repeated exposure. No occupational factor in 2006 was predictive of MDD in models 1 and 2. All psychosocial work factors (except tensions with the public) and night work in 2010 were associated with MDD in models 1, and it was also the case in model 2 for psychological demands, low decision latitude, low reward, emotional demands, ethical conflict and job insecurity. Exposure to psychological demands, low reward and emotional demands in 2006 predicted GAD in models 1, and the effect of low reward remained significant in model 2. All psychosocial work factors (except social support and role conflict), long working hours, night work, unpredictable hours and physical exposure in 2010 were associated with GAD in models 1. Psychological demands, low reward, emotional demands, role conflict, ethical conflict and job insecurity in 2010 were associated with GAD in model 2. No interaction was found between exposure in 2006 and 2010 for MDD and GAD.

**Table 4:** **Prospective associations between exposure to occupational factors in 2006 and 2010 and MDD/GAD in 2010 adjusted for covariates**

**Men and women (N = 4711)**	**MDD**				**GAD**			
	**Models 1**		**Model 2**		**Models 1**		**Model 2**	
	**OR**_**2006**_^**†**^ **95% CI**	**OR**_**2010**_^**†**^ **95% CI**	**OR**_**2006**_^**‡**^ **95% CI**	**OR**_**2010**_^**‡**^ **95% CI**	**OR**_**2006**_^**†**^ **95% CI**	**OR**_**2010**_^**†**^ **95% CI**	**OR**_**2006**_^**‡**^ **95% CI**	**OR**_**2010**_^**‡**^ **95% CI**
**Psychosocial work factors**								
High psychological demands	0.88 [0.57-1.34]	2.93*** [1.91-4.48]	0.77 [0.46-1.27]	2.00** [1.28-3.13]	1.52* [1.02-2.28]	3.61*** [2.46-5.31]	1.33 [0.87-2.05]	2.27*** [1.47-3.51]
Low decision latitude	1.11 [0.75-1.65]	2.19*** [1.50-3.20]	1.14 [0.77-1.70]	1.55* [1.01-2.38]	1.12 [0.77-1.62]	1.67** [1.17-2.39]	1.07 [0.72-1.59]	1.25 [0.83-1.89]
Low social support	0.93 [0.59-1.47]	1.58* [1.06-2.36]	0.90 [0.54-1.50]	1.09 [0.73-1.62]	1.42 [0.95-2.12]	1.19 [0.81-1.75]	1.10 [0.74-1.64]	0.83 [0.56-1.24]
Low reward	1.22 [0.85-1.75]	2.91*** [1.99-4.26]	1.37 [0.92-2.03]	2.16*** [1.47-3.18]	1.46* [1.02-2.11]	2.14*** [1.43-3.20]	1.46* [1.00-2.12]	1.70** [1.15-2.52]
Emotional demands	0.75 [0.48-1.17]	3.86*** [2.41-6.19]	0.77 [0.47-1.26]	3.16*** [1.91-5.22]	1.57* [1.06-2.31]	2.53*** [1.66-3.86]	1.26 [0.84-1.90]	1.69* [1.10-2.60]
Role conflict	0.86 [0.57-1.29]	1.56* [1.06-2.31]	0.70 [0.45-1.10]	0.99 [0.66-1.47]	1.32 [0.90-1.92]	0.95 [0.64-1.41]	0.89 [0.59-1.33]	0.56** [0.36-0.87]
Ethical conflict	0.80 [0.52-1.23]	2.20*** [1.46-3.32]	0.81 [0.51-1.27]	1.54* [1.01-2.35]	1.07 [0.74-1.55]	2.68*** [1.87-3.85]	0.83 [0.55-1.24]	1.90*** [1.31-2.78]
Tensions with the public	0.73 [0.49-1.07]	1.18 [0.79-1.75]	0.68 [0.45-1.04]	0.74 [0.48-1.14]	1.34 [0.90-1.98]	1.57* [1.05-2.37]	1.14 [0.74-1.76]	0.98 [0.62-1.53]
Job insecurity	1.29 [0.82-2.04]	2.41*** [1.60-3.64]	1.25 [0.78-2.02]	1.90** [1.23-2.93]	1.46 [0.97-2.21]	3.05*** [2.10-4.42]	1.19 [0.77-1.84]	2.30*** [1.54-3.43]
Work-life imbalance	0.90 [0.57-1.41]	1.82** [1.20-2.78]	0.91 [0.55-1.49]	1.16 [0.74-1.80]	1.38 [0.93-2.05]	2.36*** [1.60-3.50]	1.13 [0.74-1.72]	1.41 [0.90-2.22]
**Working time/hours**								
Long working hours	1.09 [0.68-1.73]	1.21 [0.76-1.93]	1.07 [0.67-1.71]	1.05 [0.66-1.67]	1.07 [0.68-1.68]	1.58* [1.02-2.43]	0.80 [0.50-1.27]	1.10 [0.70-1.74]
Night work	0.73 [0.41-1.31]	1.88* [1.10-3.19]	0.77 [0.43-1.40]	1.43 [0.81-2.53]	0.65 [0.35-1.18]	1.76* [1.04-2.98]	0.73 [0.40-1.32]	1.30 [0.75-2.26]
Shift work	1.24 [0.72-2.15]	0.99 [0.56-1.75]	1.17 [0.64-2.14]	0.96 [0.53-1.76]	0.92 [0.50-1.69]	1.05 [0.58-1.89]	1.00 [0.55-1.80]	0.79 [0.43-1.46]
Low predictability	1.13 [0.72-1.76]	0.97 [0.62-1.53]	1.06 [0.67-1.66]	0.76 [0.47-1.23]	0.84 [0.56-1.27]	2.06*** [1.42-2.99]	0.73 [0.48-1.10]	1.39 [0.95-2.04]
**Physical work factors**								
Biomechanical exposures	1.09 [0.72-1.66]	1.16 [0.78-1.73]	1.04 [0.65-1.67]	1.09 [0.68-1.75]	0.96 [0.64-1.44]	1.28 [0.85-1.92]	0.74 [0.48-1.15]	1.12 [0.72-1.74]
Physical exposures	1.09 [0.74-1.59]	1.16 [0.81-1.68]	1.09 [0.70-1.70]	0.87 [0.55-1.35]	0.91 [0.60-1.36]	1.68* [1.12-2.51]	0.82 [0.54-1.25]	1.21 [0.78-1.87]
Chemical exposures	1.00 [0.65-1.55]	0.81 [0.52-1.26]	1.01 [0.63-1.63]	0.72 [0.45-1.16]	1.24 [0.82-1.87]	0.82 [0.53-1.26]	1.26 [0.84-1.89]	0.75 [0.48-1.19]

The two binary variables for exposure to each occupational factor in 2006 (OR_2006_) and 2010 (OR_2010_) were included in the model simultaneously.

Weighted logistic regression models.

Covariates: gender, age, occupation, marital status, having a child under 3 y, social support outside work and stressful life events.

^†^Each occupational factor studied separately. ^‡^All occupational factors studied simultaneously.

Bold OR: significant at 5%.

Wald test: ***p < 0.001, **p < 0.01, *p < 0.05.

Regarding the associations between covariates and the two outcomes (model 2), female gender, low social support outside work and life events during childhood were associated with a higher risk of MDD and GAD. No association was found between age, occupation, marital status, having a child under 3 y and life events between 2002 and 2006 and MDD/GAD.

## Discussion

### Main results

The results based on models taking all occupational factors into account simultaneously showed that low reward and job insecurity predicted MDD. Psychological demands, low reward, emotional demands and job insecurity were predictive of GAD. A less conservative approach exploring each factor separately provided a higher number of predictive factors for GAD. Indeed, additional predictive factors of GAD were found: low social support, ethical conflict, tensions with the public and work-life imbalance. Dose–response associations were observed showing that the more frequent the exposure to job insecurity, the higher the risk of MDD and GAD, and the more frequent the exposure to psychological demands and low reward, the higher the risk of GAD. No effect of repeated exposure was found on MDD or GAD.

### Comparison with the literature

The comparison of our results may be difficult with previous studies that used mental health symptom scales and did not use a diagnostic interview. Indeed, the outcome is very different in nature, as symptoms are studied in the first case, and mental disorders according to clinical criteria are assessed in the second case. In addition, depression was the most studied disorder in this topic, and anxiety was studied more seldom, and still more separately from depression [19-22]. This is why most of our comparison with the literature was restricted to studies using a diagnostic interview. Around twenty studies were found in the literature, and half of them had a prospective design. In the absence of previous studies using diagnostic criteria on a specific point in our literature comparison, other studies were searched to compare our results with the literature.

Regarding the job strain model factors, we found that psychological demands (models 1 and 2) and low social support (models 1) were predictive of GAD in our study. Some previous prospective studies showed that psychological demands [20,23], low skill discretion [23], job strain [24-27] and low social support [23,25,26] predicted depressive disorders. The prospective study by Joensuu et al. [28] reported that high skill discretion was associated with a reduced risk of hospital admissions due to depressive disorders and high decision authority was associated with an elevated risk. The cross-sectional study by Stansfeld et al. [21] showed that the three job strain dimensions, demands, latitude and support, were associated with anxiety and the cross-sectional study by Wang et al. [29] reported an association between social support and anxiety. Consequently, our study may be one of the first to demonstrate prospective associations between some job strain model dimensions and anxiety.

Low reward was found to be a predictive factor of MDD and GAD in our study. Two previous studies explored effort-reward imbalance in association with mental disorders: a cross-sectional study by Clark et al. [30] reported an association between effort-reward imbalance and common mental disorders and a prospective study by Wang et al. [31] showed that effort-reward imbalance was predictive of major depressive disorder, especially among women. To our knowledge, there has been no previous prospective study exploring the effort-reward imbalance variables in association with anxiety.

Emotional demands were predictive of GAD in our study. We also found that tensions with the public were predictive of GAD (models 1). The case–control study by Wieclaw et al. [32] showed that emotional demands and working with people were associated with depression measured using first-ever clinical diagnosis made by a psychiatrist in charge of hospital or outpatient treatment. Other previous studies showed an association of person-related work or emotional demands with depressive symptoms or the use of antidepressants [33,34].

Two role stressors were explored in our study: role and ethical conflict. Ethical conflict was predictive of GAD (models 1). Previous studies reported significant associations between conflicting demands and antidepressant use [35], and between role ambiguity and sick leave of 30 days or more due to depressive disorders [36].

Job insecurity was a predictive factor of MDD and GAD (especially for men). Two prospective studies by Wang et al. [23,31] reported that job insecurity was a predictive factor of major depressive episode. Some cross-sectional studies found an association of job insecurity with major depressive episode among men [37], with both depressive and anxiety disorders [21], and with anxiety disorders for both genders and depressive disorders for men [29].

Work-life imbalance was a predictive factor of GAD (models 1). Three studies (two cross-sectional and one prospective) by Wang et al. showed that work-life imbalance was associated with major depressive disorder, mood or anxiety disorders [22,29], and that family-to-work conflict among men and work-to-family conflict among women were predictive of major depressive disorder [31].

Dose–response associations were observed between psychosocial work factors and the two mental disorders in terms of exposure frequency in our study. The risk of MDD and GAD increased with the frequency of exposure to job insecurity and the risk of GAD increased with the frequency of exposure to psychological demands and low reward. However, no effect of repeated exposure was found on MDD or GAD. The literature is scarce on this topic. We found three prospective studies using diagnostic interview that explored the effects of 2 or 3 measures of exposure on major depressive episode in the literature. These studies demonstrated that repeated exposure to job strain and change from non-exposure to exposure to job strain predicted depression [25,26,38]. To our knowledge, no previous study was performed on the association between repeated exposure to psychosocial work factors and anxiety.

### Strengths and limitations

The strengths of the study may be underlined. The study was based on a large national representative sample of the French population (i.e. not a specific or selected population of workers) with satisfactory response and follow-up rates. A comparison was performed between respondents and non-respondents in 2010 among the whole sample of workers in 2006 and showed that non-respondents were more likely to be younger and without partner. As weighted data (taking amongst others, age and size of household, into account as calibration variables) were used in all analyses, we controlled for potential biases related to non-response and attrition and the results may be generalized to the whole national population. We found gender differences in the prevalence of mental disorders, occupational factors and covariates and we examined potential gender differences in the associations between occupational factors and mental disorders. However, only a very small number of interactions were observed suggesting that most of these associations may be similar for both genders [39]. The study design was prospective, making clear the temporal sequence between exposure to psychosocial work factors and incidence of mental disorders. Mental disorders were measured using a diagnostic interview and both depression and anxiety were explored. Psychosocial work factors were examined including classical concepts (from the job strain and effort-reward imbalance models) as well as understudied concepts (emotional demands, role stressors, tension with the public, job insecurity, work-life imbalance). Exposure frequency and repeated exposure were studied, bringing some elements on dose–response associations. Two types of models were performed allowing us to study the associations between psychosocial work factors and mental disorders using two approaches, the first one exploring each factor separately, and the second one exploring all factors together, i.e. independently of each other. The second approach may be considered conservative and lead to overadjustment as there may be complex interrelations between factors, and factors may be causes or consequences of other factors [40]. Major covariates, considered as well-known risk factors of mental disorders, were taken into account, and their results were consistent with the literature. Furthermore, sensitivity analyses were done and the results were unchanged.

Some limitations deserve to be mentioned. Although the study design was prospective, the follow-up was performed four years after the baseline collection of data. Consequently, changes in exposures and/or outcome may not have been captured properly, and may lead to exposure misclassification and underestimation of the associations. This may contribute to explain the non-significant results found for repeated exposure. In particular, a healthy worker effect may be suspected as workers developing mental disorders within the 4-year period may have left the labour market in 2010. A sensitivity analysis was performed including major changes in job and working conditions and long non-working period(s) between 2006–2010 as additional covariates and the results were unchanged. Furthermore, a sensitivity analysis including all people at follow-up whatever their working status in 2010 (and not only those who were working) also provided similar results. This analysis also showed that the change from working status in 2006 to non-working status (for other reasons than retirement) in 2010 increased the risk of both disorders. A reporting bias may be suspected as both exposure to psychosocial work factors and mental disorders were self-reported. Nevertheless, it may be assumed that this issue may be reduced as a diagnostic interview was used. Furthermore, a sensitivity analysis was performed to control for overcommitment that may be considered as a personality factor and the results were not modified, and overcommitment was found to be a predictive factor of both disorders. Psychosocial work factors were not measured using validated questionnaires and most of them were based on one single item, leading to potential imprecision in the variables used. However, other authors underlined the interest and validity to construct proxies [41]. Some psychosocial work factors were neglected as they were not available in the survey, and may be important in the association with mental disorders such as organizational justice [42].

## Conclusions

This study suggests that both classical and emergent psychosocial work factors may increase the risk of depression and anxiety with dose–response associations in terms of exposure frequency. More research may be needed to confirm these results using prospective design, diagnostic interview and sophisticated measures of exposure. Prevention policies oriented toward psychosocial work environment comprehensively may be useful to improve mental health at the workplace.
